# Investigation of resistance against to flumethrin using against *Varroa destructor* in Türkiye

**DOI:** 10.1007/s11259-024-10351-x

**Published:** 2024-03-21

**Authors:** Ender Yarsan, Fatih Yilmaz, Sedat Sevin, Gökhan Akdeniz, Bekir Celebi, Seyit Hasan Ozturk, Sultan Nurhan Ayikol, Umit Karatas, Hasan Ese, Nuri Fidan, Bayram Agacdiken, Cahit Babur, Mucahit Buldag, Sinem Pehlivan

**Affiliations:** 1 Department of Pharmacology and Toxicology, Faculty of Veterinary Medicine, Ankara University, Ankara, 06100 Türkiye; 2 Apiculture Research Institute, Ordu, Türkiye; 3 Apiculture Research Center, Aegean Agricultural Research Institute, İzmir, Türkiye; 4 Microbiology Reference Laboratory Department, General Directorate of Public Health, Ministry of Health, Ankara, Türkiye; 5 Food Control Laboratory Directorate, Giresun, Türkiye; 6 Ordu-Kabadüz District Directorate of Agriculture and Forestry, Ordu, Türkiye; 7 Department of Medical Pharmacology, Faculty of Medicine, Ankara Medipol University, Ankara, Türkiye

**Keywords:** Flumethrin, Honey bee, Mutation, Resistance, Sequencing, *Varroa destructor*

## Abstract

The honeybee ectoparasite Varroa destructor is a major threat to apiculture when evaluating bee diseases and pests. While attempting to control this mite, beekeepers often depend on a small selection of authorized synthetic acaricides, such as flumethrin, which is widely used in Türkiye and globally. However, resistance to flumethrin develops due to incorrect and excessive use. In this study conducted at Ordu Beekeeping Research Institute, trial group were established including an untreated control group and group where flumethrin-based pesticides were applied. Dead varroas collected from pollen traps and live varroas collected from bees were obtained from these trial groups for molecular analysis as positive-negative controls. Varroa samples were collected from provinces representing different regions with intensive beekeeping activities such as Adana, Ankara, Bingöl, Muğla, Ordu, Şanlıurfa, Tekirdağ. Molecular methods were employed to investigate the resistance gene region for pyrethroids (specifically flumethrin) against *V. destructor*. In our study, individual DNA extractions were performed on dead parasites from colonies subjected to pyrethroid application (resistance negative control) and live parasites (resistance positive control). The DNA samples obtained were used in PCR reactions targeting the region encoding the 925th amino acid of the voltage-gated sodium channel (VGSC) gene, which is responsible for resistance formation. The DNA samples were subjected to gel electrophoresis to observe the amplification products of the expected target region. To examine the nucleotide sequence changes that encode leucine at the 925th amino acid, which is associated with resistance, DNA sequence analysis was applied to the amplification products. Out of 332 *V. destructor* parasites obtained from different provinces, 279 were analysed using molecular methods. It was observed that 31% of the samples showed sensitivity to flumethrin while 69% exhibited resistance to it. Among the resistant samples: 27% had homozygous isoleucine mutation; 28% had homozygous valine mutation; 2.8% had heterozygous isoleucine mutation; 8.5% had heterozygous valine mutation; and 2.8% had heterozygous methionine mutation, all of which were associated with flumethrin resistance. As a result, the rate of flumethrin resistance in parasites varied between 51% and 94% among different provinces.

## Introduction

Hailing from Asia and Europe, honeybees and beekeeping possess a significant role in ecology, economy and ensuring food security (Gallai et al. 2009) In a general sense, apiculture is an important agricultural activity encompassing the production of various products such as honey, beeswax, pollen, propolis, royal jelly, bee venom, and living materials like queen bees, swarms, and package bees. Apiculture, a practice carried out worldwide and in our country for many years, contributing to plant pollination of agricultural crops and wild flora. Apiculture, in addition to all these mentioned benefits, is also a livelihood that possesses significant advantages, such as the ability to be conducted with minimal labor and capital, without being tied to a specific piece of land. Due to these advantages, apiculture has been a significant pursuit for people in our country and around the world since ancient times (Doğanay and Aydın 2017; Gregor et al. 2022). The most effective method recommended for combating Varroa infestations is through the use of pesticides; however, it is not possible to assert that the drugs/chemotherapeutic agents used in this context are entirely effective. Therefore, colonies should be treated with pesticides at specified intervals and in accordance with the instructions provided in the product label through a proper program. On the other hand, the risk of developing resistance to the pesticides used for Varroa control is a highly significant concern. Resistance development is encouraged by several factors, such as applying drugs in dosages significantly lower or higher than recommended by the manufacturer, keeping the preparations in colonies for longer periods than advised, and repeatedly using expired drugs (Rinkevich et al. 2017; Gregor et al. 2022). Unfortunately, such errors are often made by beekeepers for economic reasons, unaware that short-term savings can lead to long-term costs. Furthermore, the resistance of Varroa to acaricides may increase as residues of the relevant drugs accumulate in bee colony products, particularly in beeswax (Bak ve ark., 2012; Rinkevich 2020). In recent times, there has been an increasing occurrence of colony collapse disease due to the growing resistance of Varroa mites to existing pesticides. During the initial stages of their use, newly formulated acaricides exhibit high efficacy. However, shortly after the introduction of these acaricides, the rapid adaptability of Varroa mites results in the emergence of acarids that are resistant to the acaricides and capable of reproducing (Mathieu and Faucon 2000; Vlogiannitis et al. 2021a, b). As the number of Varroa generations developing in the presence of an acaricide increases, the percentage of the population becoming resistant to the acaricide also rises over time. This phenomenon is a consequence of the enhanced synthesis and release of detoxification enzymes in Varroa. In Europe, synthetic pyrethroids are the group of pesticides that exhibit the highest effectiveness against Varroa. Resistance to pyrethroids arises from an increase in the activity of the monooxygenase enzyme in the cytochrome P-450 system of Varroa mites (Watkins 1997; Hernández-Rodríguez et al. 2021). Among the synthetic pyrethroids used, Tau-fluvalinate and flumethrin are common. However, it has been reported that their effectiveness has decreased due to the development of resistance (Trouiller 1998; Lipiński and Żółtowska 2005; Goodwin et al. 2005; Erdem et al. 2024). In Italy, losses attributed to mite resistance have been reported to be over 70% (Bak et al. 2012). High levels of resistance have also developed against other pesticides used in Varroa control besides pyrethroids (Akkaya and Vurusaner 1997; Mitton et al.,^2022^). When reviewing literature studies, it becomes evident that Varroa resistance has been increasing steadily over time. In our study, varroa samples were collected from seven different regions of Türkiye and the resistance status was investigated.

## Materials and methods

### Determination of positive and negative resistance groups for *Varroa destructor*

Within the scope of the study, flumethrin preparation licensed by the Turkish Ministry of Agriculture and Forestry was used in the apiary area of the Ordu Beekeeping Research Institute Directorate to determine positive and negative controls for Varroa agents. The hives of the Beekeeping Institute are treated alternately with preparations containing the active ingredients amitraz and flumethrin in the fight against varroa. Application was made with strip-shaped flumethrin preparation in 10 hives. Varroa falling on the hive bottom board after 1 week (negative control); Varroa (positive control) collected by the powdered sugar method (Muz et al. 2014) were shipped to laboratories under cold chain to be used in molecular studies. DNA Extraction, PCR Amplification, and Sequence Analysis were performed in this laboratory.

### Collection of Varroa samples in field conditions

**Fig. 1:**
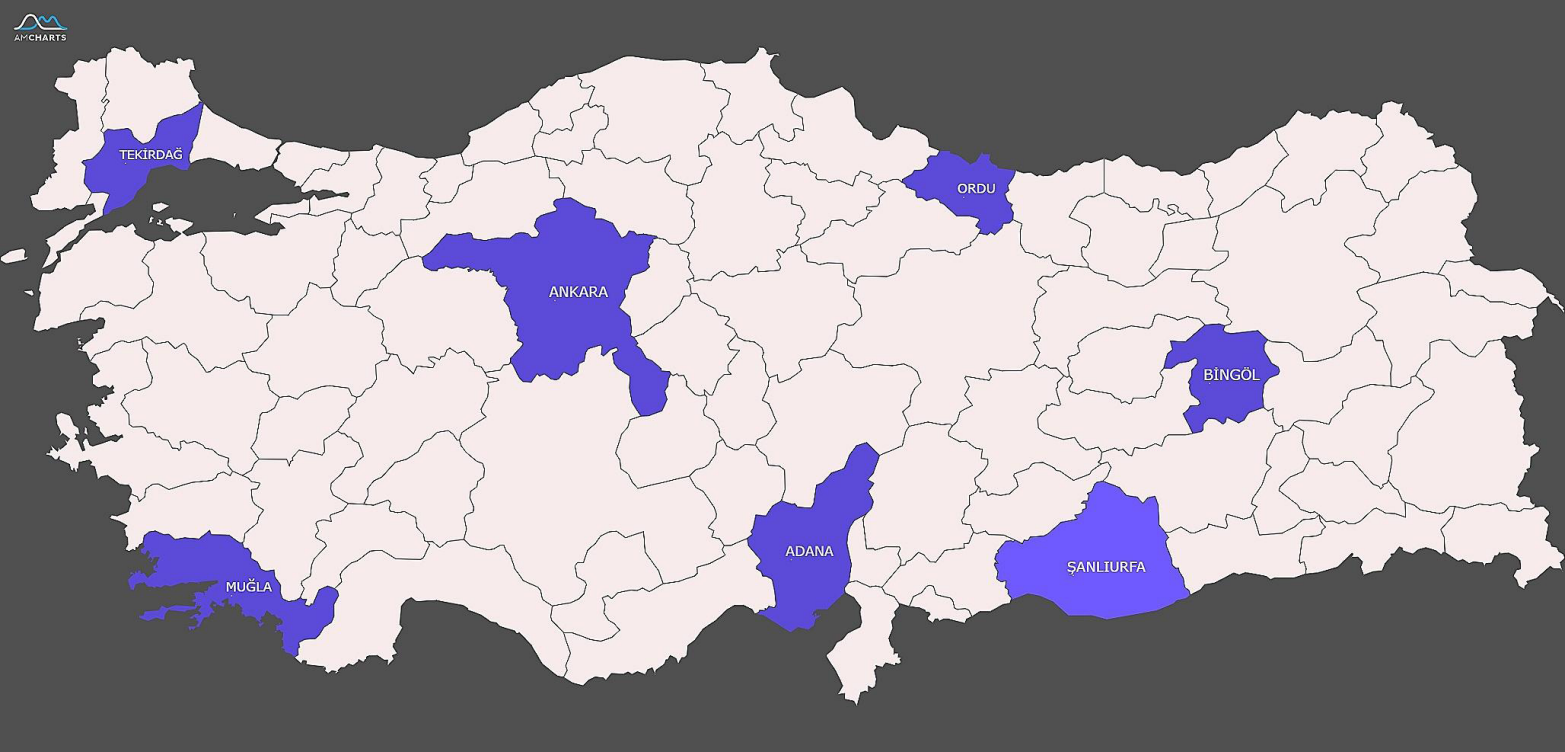
Selected provinces; Adana, Ankara, Bingöl, Muğla, Ordu, Şanlıurfa and Tekirdağ

Flumethrin resistance in Varroa mites has been molecularly investigated based on epidemiological studies conducted in seven different regions and selected provinces; Adana, Muğla, Ordu, Ankara, Tekirdağ, Bingöl, and Şanlıurfa (Fig. 1), forming the basis of the study.

Samples were collected in September-October 2021. The sampled hives were chosen randomly. At least 50 Varroa samples were analyzed from each region. Varroa agents were collected from a wide range of areas within each province. The selected provinces were specifically chosen as focal points for beekeeping activities in our country. The obtained samples were promptly delivered to the laboratory, and Varroa mites were collected from the colonies using the powdered sugar method (Muz et al. 2014). The resistance status of these samples was investigated using molecular techniques. For this purpose, previously selected positive and negative controls provided guidance and formed the basis for the evaluations.

### DNA extraction, PCR amplification, and sequence analysis

Individually, adult parasites were dissected into small pieces using a scalpel and placed into 2 ml Eppendorf tubes. To each tube, 150 µl of PBS was added, and DNA extraction was performed. For DNA extraction, the Qiagen QIAamp mini kit (Hilden, Germany) was used following the manufacturer’s instructions. The obtained DNA samples were stored at -20 °C until further use.

To determine resistance to pyrethroids in *V. destructor* by identifying nucleotide changes at leucine (CTG) at amino acid position 925 in segment 5 of the voltage-gated sodium channel (VGSC) gene, conventional PCR was conducted using the primers Vd_kdr_F1 (5´GAGTCTTCAAACTAGCCAAG 3´) and Vd_kdr_R1 (5´ACACTTGTTGTCGAGATAGT 3´) as described by Alissandrakis et al. (2017).

Amplitaq Gold Master Mix (Applied Biosystems, Foster City, CA, USA) was used for PCR, with 10 picomoles of forward and reverse primers added to each reaction. Genomic DNA from the samples was added to a final reaction volume of 5 µl, and the PCR protocol described by Alissandrakis et al. (2017) was followed: an initial denaturation at 95 °C for 3 min, followed by 35 cycles of denaturation at 95 °C for 30 s, annealing at 55 °C for 30 s, extension at 72 °C for 30 s, and a final extension at 72 °C for 2 min. PCR products were run on a 2% agarose gel, and the expected approximately 170-base pair amplification products were observed. Positive amplification products were sent for DNA sequence analysis.

For DNA sequence analysis, the PCR amplification products were purified using the “ExoSAP-IT™ PCR Product Cleanup Reagent” kit (Thermo Fisher Scientific, USA) following their procedures. DNA sequencing was performed using the ABI 3730XL Sanger sequencing instrument (Applied Biosystems, Foster City, CA) and the BigDye Terminator v3.1 Cycle Sequencing Kit (Applied Biosystems, Foster City, CA). The obtained sequences were analyzed using FinchTV 1.4 software.

## Results

**Fig. 2:**
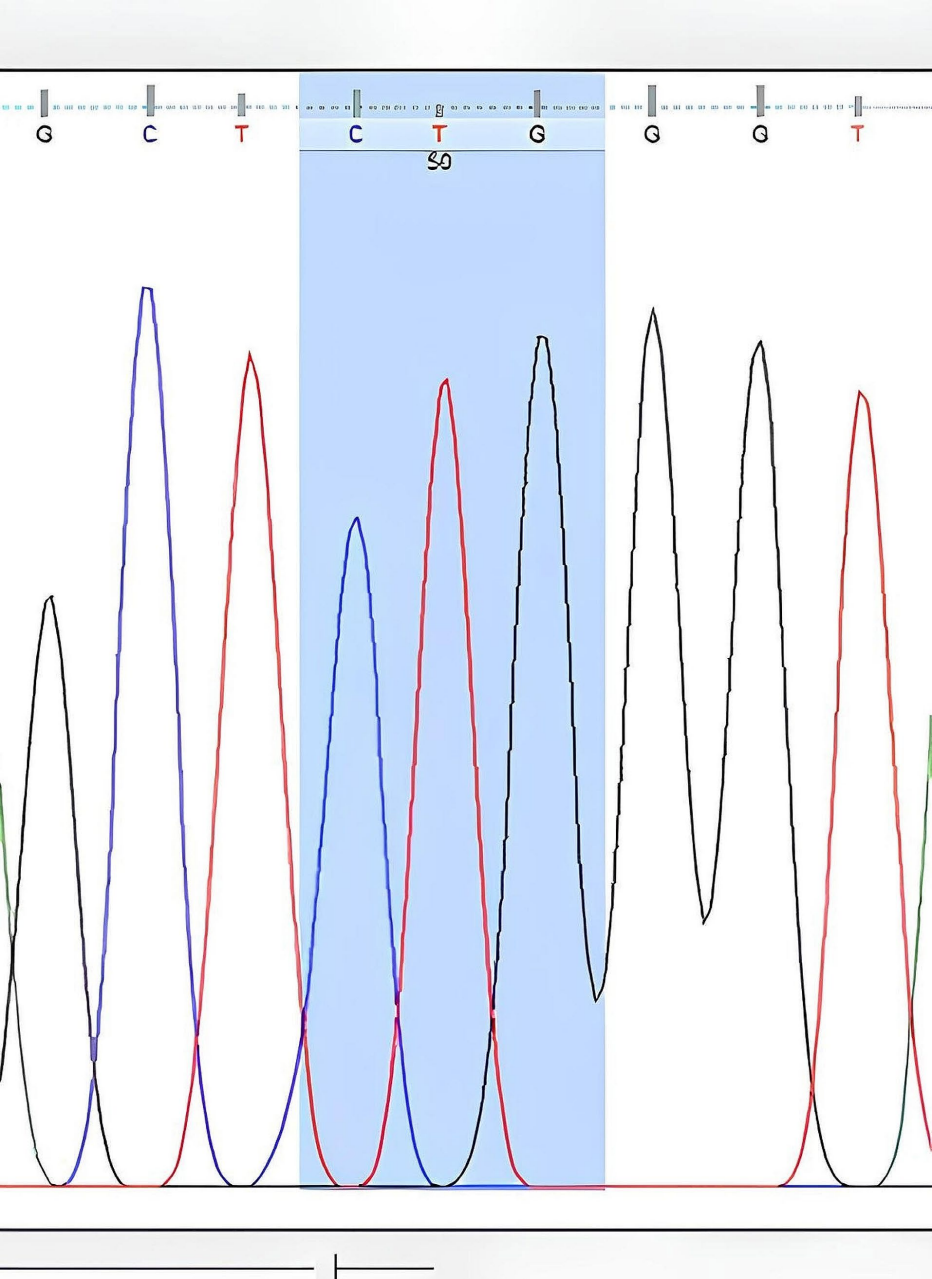
Nucleotide sequence of leucine (CTG) at the 925th amino acid of the *V. destructor* (VGSC) gene (Homozygous sensitive individual)

**Fig. 3:**
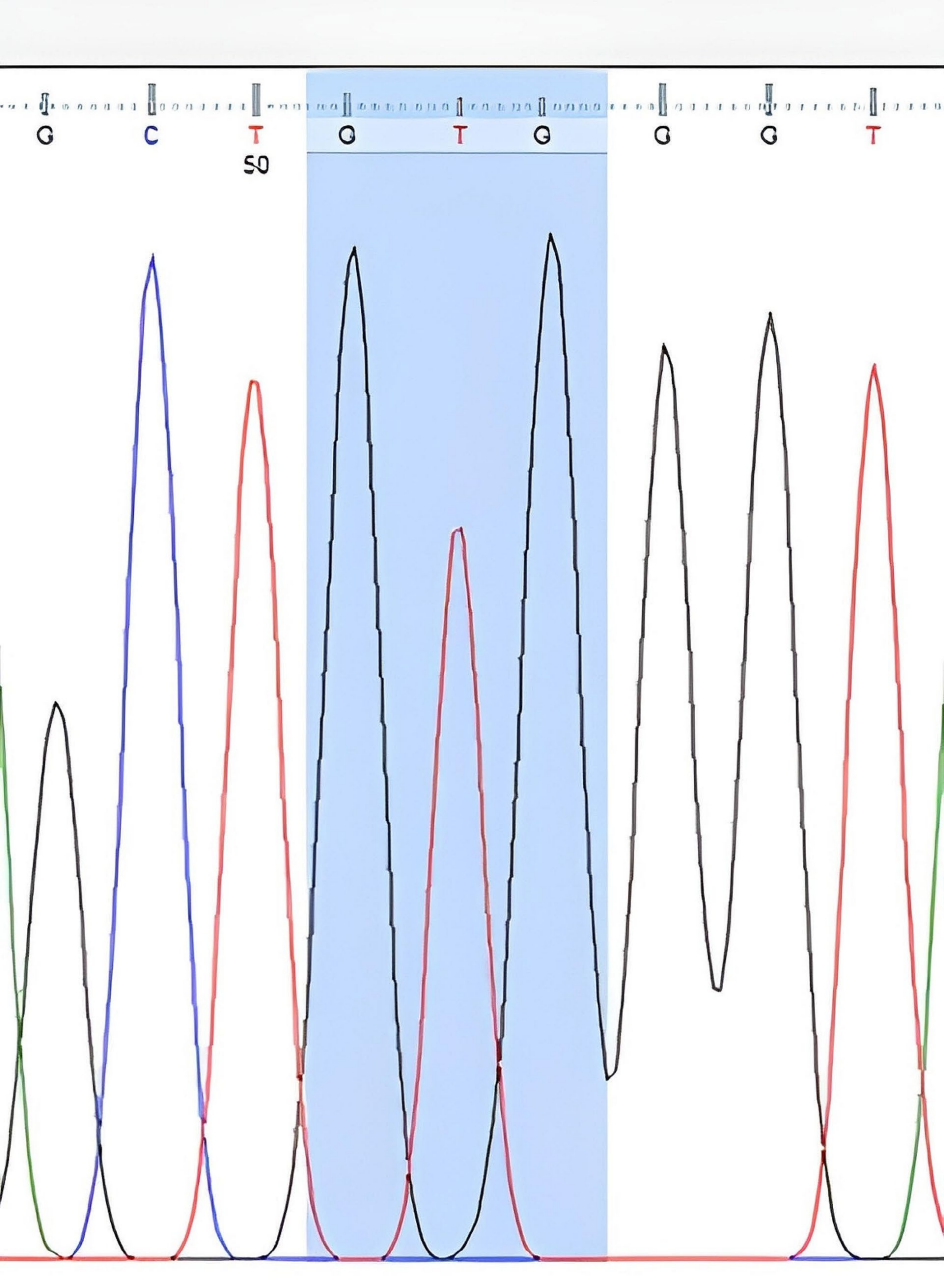
Nucleotide sequence of valine (GTG) at the 925th amino acid of the *V. destructor* (VGSC) gene (Homozygous resistant individual)

**Fig. 4:**
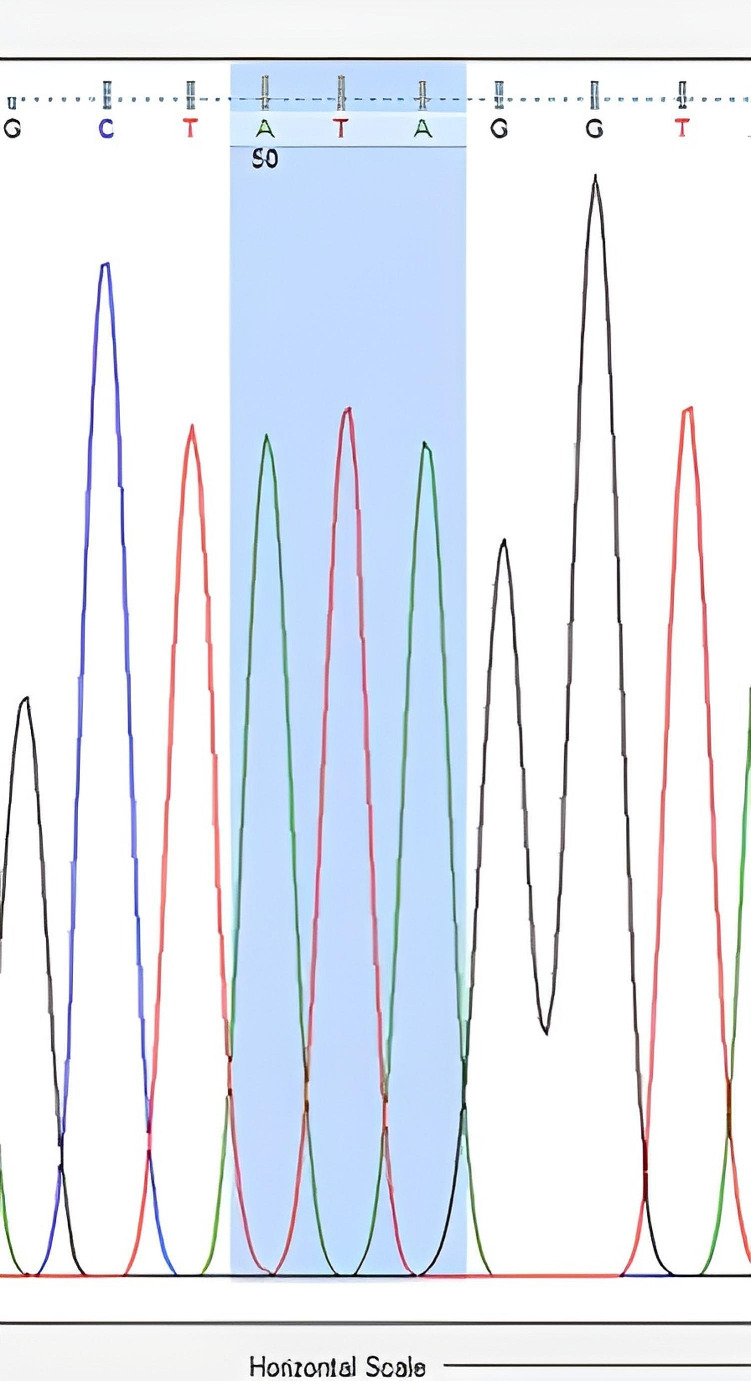
Nucleotide sequence of isoleucine (ATA) at the 925th amino acid of the *V. destructor* (VGSC) gene (Homozygous resistant individual)

**Fig. 5:**
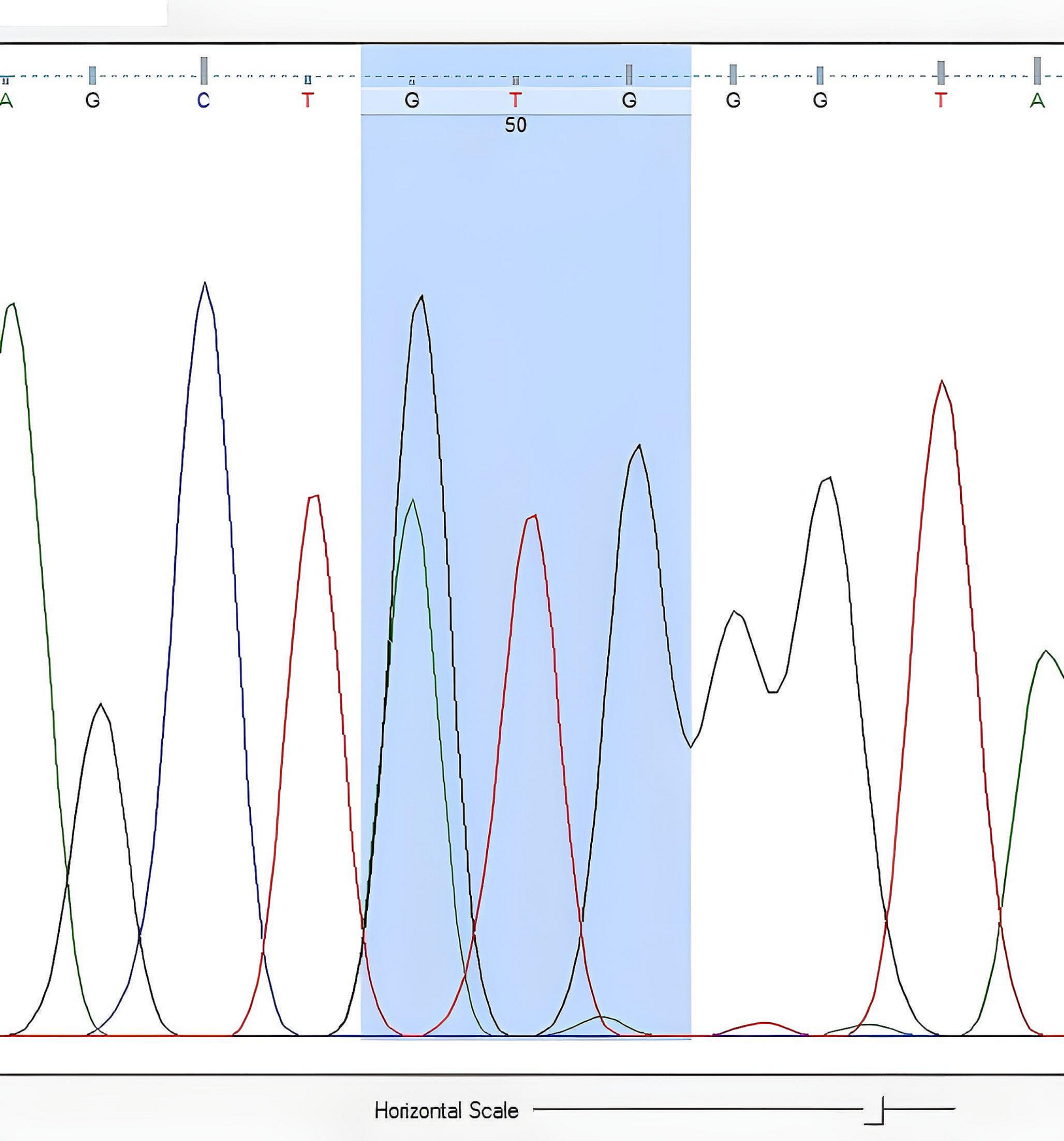
Nucleotide sequence of valine/methionine (GTG/ATG) at the 925th amino acid of the *V. destructor* (VGSC) gene (Heterozygous resistant individual)

The nucleotide sequences obtained from DNA sequence analysis were compared with GenBank data, and it was determined that the obtained sequences matched the *V. destructor* (VGSC) gene. The nucleotide sequences encoding leucine at the 925th amino acid were found based on the amino acid sequence. While CTG sequences were observed in resistance-negative samples (Fig. 2), mutations resulting in valine (GTG) (Fig. 3) and isoleucine (ATA) (Fig. 4) due to C-G substitutions, as well as mutations resulting in isoleucine (ATA) due to C-A/G-A substitutions, were observed in many samples. A third mutation in this region, C-A substitution resulting in methionine (ATG), was also determined (Fig. 5).

**Fig. 6:**
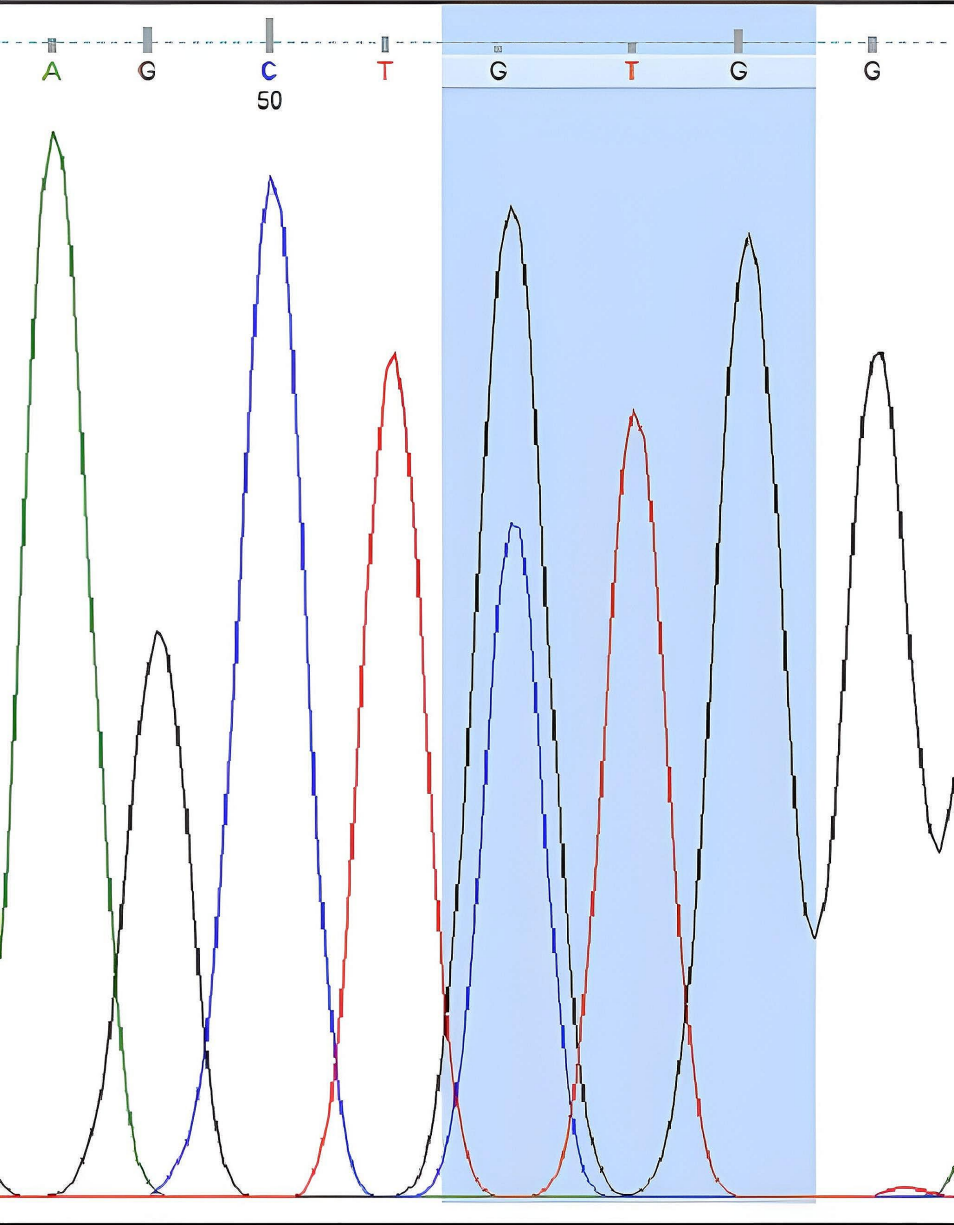
Nucleotide sequence of valine/leucine (GTG/CTG) at the 925th amino acid of the *V. destructor* (VGSC) gene (Heterozygous resistant individual)

**Fig. 7:**
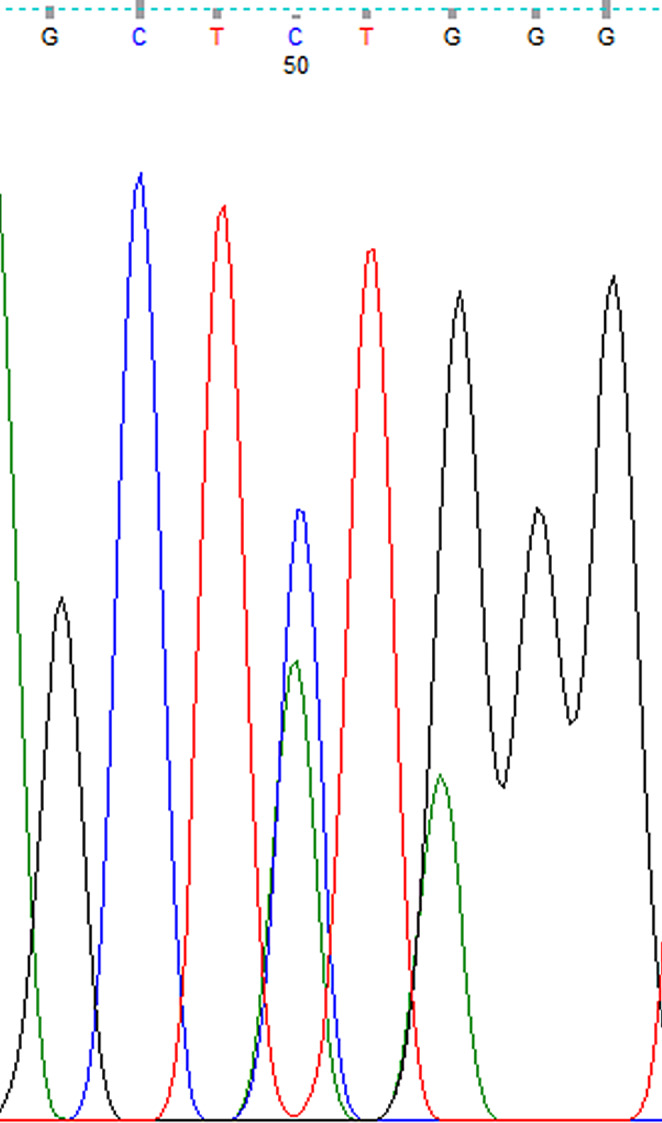
Nucleotide sequence of leucine/isoleucine (CTG/ATA) at the 925th amino acid of the *V. destructor* (VGSC) gene (Heterozygous resistant individual)

DNA sequence analysis revealed the CTG nucleotide sequence encoding leucine at the 925th amino acid in sensitive individuals (Fig. 2). In resistant individuals, the nucleotide sequences encoding valine (GTG) and isoleucine (ATA) were identified (Figs. 3 and 4). If an individual received a resistant gene from both parents, the observed nucleotide sequence was considered homozygous, as shown in Figs. 3 and 4. If an individual received a resistant gene from one parent and a sensitive gene from the other parent, it was considered heterozygous (Figs. 6 and 7-5).

**Table 1:** Distribution of flumethrin resistance in *V. destructor* parasites (VGSC gene) by province

				RESISTANCE TO ISOLEUCINE		RESISTANCE TO VALINE		RESISTANCE TO METHIONINE		
PROVINCES	SAMPLED NUMBER (n)	Sequenced Sample Number (n)	Sensitive Individuals (n) %	Homozygous Individuals (n) %	Heterozygous Individuals (n) %	Homozygous Individuals (n) %	Heterozygous Individuals (n) %	Homozygous Individuals (n) %	Heterozygous Individuals (n) %	TOTAL RESISTANCE (n) %
ADANA	46	45	9 20%	20 44%	3 7%	8 18%	1 2%		4 9%	**36** **80**%
ANKARA	70	68	28 41%	23 34%	5 7%	11 16%	1 1.4%			**40** **59**%
BİNGÖL	48	45	22 49%	1 2%		13 29%	9 20%			**23** **51**%
MUĞLA	24	18	2 11%	16 89%						**16** **89**%
ORDU	50	32	2 6%	7 %2%		17 53%	6 19%			**30** **94**%
TEKİRDAĞ	48	40	14 35%	5 12.5%		18 45%	3 7.5%			**26** **65**%
ŞANLIURFA	46	31	9 29%	3 10%		11 35%	4 13%		4 13%	**22** **71**%
**TOTAL**	332	279	**86** **31**%	**75** **27**%	**8** **2.8**%	**78** **28**%	**24** **8.5**%		**8** **2.8**%	**193** **69**%

**Fig. 8:**
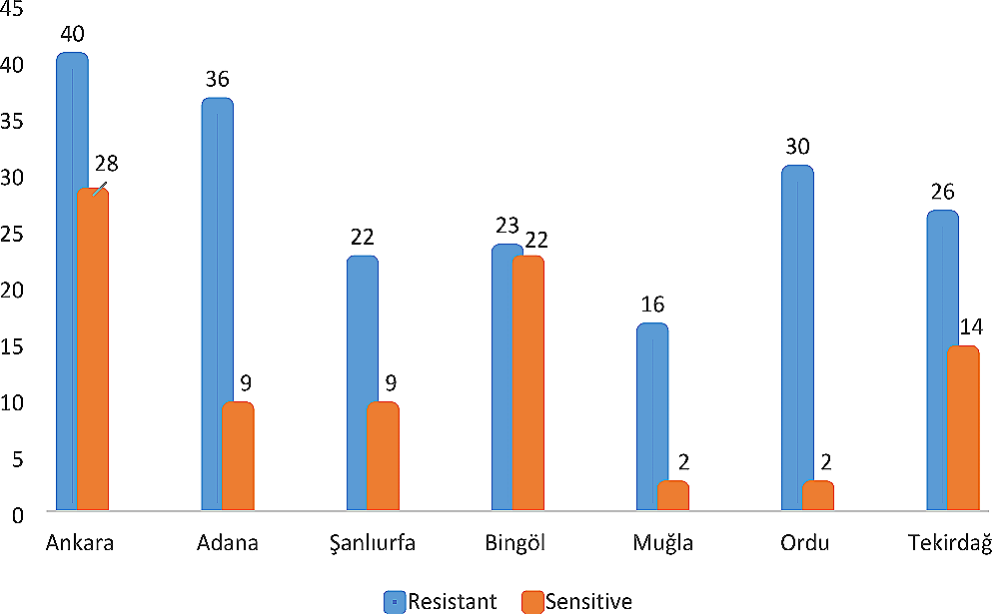
Distribution of susceptibility/resistance of *V. destructor* parasites to flumetrin by province

DNA extraction was performed on 332 *V. destructor* parasites obtained from different provinces, and target region amplification was carried out through PCR. Among the 279 obtained samples, 86 (31%) exhibited the CTG (leucine) sequence, indicating sensitivity, while 75 (27%) displayed homozygous ATA (isoleucine), 78 (28%) showed homozygous GTG (valine), 8 (2.8%) had heterozygous ATA/CTG (isoleucine heterozygous), 24 (8.5%) presented heterozygous GTG/CTG (valine heterozygous), and 8 (2.8%) exhibited heterozygous ATG/CTG (methionine heterozygous) nucleotide sequences, indicating resistance, as shown in Table 1. The highest resistance rate, 94%, was observed in parasites collected from Ordu province, while the lowest resistance rate, 51%, was observed in parasites collected from Bingöl province. The resistance and sensitivity rates by provinces are provided in Table 1; Fig. 8.

## Discussion

In the European context, the L925V mutation has been identified in regions more than 1000 km apart, including the United Kingdom. This suggests that the mutation has spread throughout the continent, allowing the assumption that the modification of this target region is the primary mechanism of pyrethroid resistance in *V. destructor* populations in Europe (González-Cabrera et al. 2018). In the United States, the most observed mutations are L925M and L925I (González-Cabrera et al. 2016). Although the most prevalent mutation in Europe is L925V, recent reports have indicated the presence of L925I substitution in Greece, Türkiye, and Belgium (Alissandrakis et al. 2017). In South America, the L925V mutation has been associated with flumethrin resistance (Vlogiannitis et al. 2021a, b). Alissandrakis et al. (2017) conducted a study in Greece, where they investigated the presence of resistance mutations in the voltage-gated sodium channel (VGSC) gene. They identified two amino acid substitutions, leucine-valine (L925V) and leucine-isoleucine (L925I), at the 925th position of the VGSC gene in the IIS4-IIS5 region. They also reported the high frequency of pyrethroid-resistant mutations in V. destructor populations in Greece.

Millán-Leiva et al. (2021a) conducted a study in which they collected samples from 60 locations in 12 different countries to investigate the relationship between the M918L base change associated with pyrethroid resistance in other species and L925V in V. destructor. They reported that the M918L mutation, associated with resistance to tau-fluvalinate, was reported for the first time in V. destructor in Spain, along with the presence of L925V. Phylogenetic analysis supported the independent origin hypothesis for resistance alleles in the United States and Europe, and a close relationship was observed between the L925V and L925I alleles. The same researchers (Millán-Leiva et al. 2021b) also conducted a study in which they examined the samples collected from bee colonies in 2016 and 2017. They reported that the L925V mutation was not detected in the United States; instead, L925I and L925M mutations were prevalent. They also noted that the leucine allele at position 925 of VGSC was dominant and consistently found at the same frequency in samples collected every two years (54.7%). Hernandez et al. (2021) conducted a study in the Comunitat Valenciana region of Spain, which consists of three provinces with a large professional beekeeping sector and intensive migratory beekeeping activities. They aimed to determine the effectiveness of pyrethroids, coumaphos, and amitraz-based acaricide treatments against V. destructor using TaqMan® genotyping tests. The frequency of pyrethroid-resistant and susceptible mites was determined for each sample after genotyping 40 individual mites for the presence of L925V mutations in VGSC. When TaqMan tests were performed, a wide range of allele frequency patterns was found in bee colonies evaluated in both 2018 (41%) and 2019 (36%). The effectiveness of different acaricides in each bee colony was not significantly associated with the geographical location across the region. Coumaphos and pyrethroids showed the highest variation in effectiveness and were often less effective in many bee colonies. Li et al. (2022) conducted a study in which they genotyped Varroa mites collected from different geographic regions of Portugal between April and August 2019 using PCR-RFLP. They found that 47% of the sampled mites carried mutations associated with resistance. González-Cabrera et al. (2018) conducted extensive genotyping of mites collected from various locations across Europe. A total of 3398 Varroa samples from Austria, Belgium, Russia, Germany, Hungary, Italy, Spain, the Netherlands, and the United Kingdom were analyzed. The study revealed the widespread distribution of L925V throughout the continent, with most countries having mites carrying the mutation, albeit at varying frequencies. The mutant mites were mostly homozygous for the resistant allele, with very few heterozygous individuals. Stara et al. (2019) used tau-fluvalinate to detect resistance to tau-fluvalinate in Varroa mites. They found that resistance values corresponding to the density of the resistant allele were associated with mite survival. In bottle tests, the survival rate of the control group (70.4%) was significantly higher than that of the group treated with tau-fluvalinate (34.3%). Mite survival was significantly correlated with the average resistance ratio. After tau-fluvalinate application, the individuals that died exhibited an average resistance value of 0.0783, while survivors showed an average resistance value of 0.400.

Koç et al. (2021) tested the resistance status of 22 V. destructor populations collected from Muğla, Ordu, Eskişehir, Zonguldak, and Ankara. The study revealed that, except for the Polatlı population, all other populations harbored one or a combination of the L925V and L925I mutations, and the L925M mutation was not detected. Different levels of the L925V and L925I mutations associated with resistance were found in over 75% of the investigated Varroa samples.

Almecija et al. (2022) showed that a high correlation of 0.89 was observed between the in vitro phenotypic test for tau-fluvalinate resistance and the presence of the L925V mutation in *V. destructor* mites in French populations. This suggests that the L925V mutation primarily accounts for resistance to tau-fluvalinate in these populations, although other mechanisms may also contribute to mite survival in the phenotypic test.

Another factor contributing to the development of resistance in Varroa ectoparasites against synthetic pyrethroids is their residue in beeswax. Medici et al. (2015) reported that the use of commercial wax mixed with paraffin and other olefins in the production of wax foundation sheets is a common practice in Argentina. They aimed to determine the presence of contaminants and acaricide residues in commercial wax used in Argentina. They also investigated the relationship between the coumaphos content in beeswax and the development of acaricide resistance reported in recent years in Argentina. The results showed that paraffin was the most common contaminant in both recycled and commercial beeswax used for wax foundation sheets in the country. Coumaphos was the most common acaricide found in beeswax, with 87% of commercial wax samples and 80% of recycled wax samples containing it. Fluvalinate was detected in 33% of commercial wax samples and 27% of recycled wax samples. A positive relationship was established between coumaphos residues in beeswax and the development of varroa resistance.

Benito-Murcia et al. (2021) discovered the chronic presence of tau-fluvalinate residues in beeswax and bee bread obtained from colonies not treated with tau-fluvalinate for four years.

Despite numerous reported cases of resistance to synthetic pyrethroids in Varroa mites, their continued intensive use in the fight against this ectoparasite persists. Previous studies supporting our findings have demonstrated that there are three different mutations (L925V, I, and M) associated with resistance at the 925th amino acid position in the voltage-gated sodium channel of *V. destructor*.

As observed in Spain, research conducted between 2006 and 2021 examined the presence of pyrethroid resistance mutations, particularly in the voltage-gated sodium channel (VGSC) gene. The research identified the L925V and M918L mutations in this gene, with a notable observation that the M918L mutation was consistently found in combination with L925V, and both mutations were homozygous. This combination of mutations seems to confer higher resistance to pyrethroids compared to the L925V mutation alone, indicating an increasing challenge in controlling pyrethroid-resistant Varroa mites (Benito-Murcia et al. 2022).

In the study investigating the threat posed by varroa to honey bee populations in New Zealand between 2017 and 2021, it was determined that the main cause of colony losses in 2021 was the varroa parasite. While the LC50 value observed in a trial conducted in New Zealand in 2003 (Goodwin et al. 2005) was 12 µg/g, this value increased 13-fold in 2022, and the adjusted LC50 value increased to 156 µg/g (McGruddy et al. 2023). RCV bioassay of 14 field mite populations collected in Korea in 2021 showed potential development of resistance in four populations. As an alternative approach, quantitative sequencing was used to evaluate the frequency of the L925I/M mutation in the voltage-gated sodium channel (VGSC) associated with the fluvalinate kdr trait. Considering that Coumaphos demonstrated significantly higher efficacy (approximately 438-fold) than fluvalinate based on LC50 values and has not been used in Korea for over two decades, it was determined to be a suitable option for the management of fluvalinate-resistant Varroa mite populations (Lee et al. 2023). In the Czech Republic, it has been reported that odor recognition and oxidative stress pathways in varroa parasitized honey bees are inhibited compared to unparasitized honey bees, the behavioral immune system of varroa parasitized honey bees is impaired, and the parasites spread faster in colonies (Kunc et al. 2023). 69% of Varroa mites collected from different regions of France exhibited the homozygous mutant genotype following final treatment with tau-fluvalinate (< 2 years) (Almecija et al. 2022). In a study conducted in five different regions in Canada, namely east, central, Niagara, northwest and southwest, amitraz was found to be “mostly effective” (90–97%) in the fight against the Varroa parasite, while flumethrin and tau-fluvalinate were found to be effective against V. destructor, which was evaluated in this study. It has been reported to show “minimal effectiveness” (< 80%) in populations (Morfin et al. 2022). Resistance evaluation was carried out using the PCR-RFLP method on 96 varroa parasites collected from different geographical regions of Portugal. 47% of mites sampled exhibited correlated mutations. The samples show predominantly homozygosity, which indicates that the mites are a highly inbred population (Li et al. 2022).

The observed resistance status, which emerged in various mutations in 69% of the 193 samples in our study, underscores the gravity of the issue. Furthermore, the occurrence of the methionine mutation in only 2 out of the 7 sampled provinces and its heterozygous resistance status may be considered an indicator that this mutation is relatively new in our country.

In studies on varroa mite resistance to pyrethroids, it is noteworthy that the tested mites are mostly collected by beekeepers. It can be observed that almost all of these studies lack a well-designed sampling plan and reliable information regarding the treatment history of colonies. Despite our knowledge that the presence of the mutation correlates with the recent history of colonies being treated with pyrethroids ((González-Cabrera et al. 2016), determining the relationship between the frequencies of genotypes obtained from colonies with unknown treatment histories and the overall resistance status of *V. destructor* populations in any country remains challenging.

Organic acids, having a similar level of efficacy to pyrethroids, can contribute to increasing the success of control when used in conjunction with pyrethroids, reducing pyrethroid usage, and preventing resistance development. Considering the resistance status observed in the studied active substance, it is evident that determining resistance mechanisms in other active substances used in varroa control is necessary. The availability of phenotypic and molecular tests plays a significant role in the application of prophylactic treatments, the development of resistance management strategies, and monitoring. In addition to developing new-generation drugs for varroa control, including pyrethroids, it should be emphasized as a crucial step for the sector to establish a proactive monitoring program involving rotations (Qadir et al. 2021).

*V. destructor* exhibits a haplo-diploid sex determination system and high levels of inbreeding, facilitating the rapid emergence of advantageous mutations and the production of homozygous individuals in the population. Accumulation of acaricide residues in beeswax, especially in different developmental stages of bees, has adverse effects on honey bees. Particularly, the long history of treatments with pyrethroid-based acaricides and their accumulation in beeswax should not be overlooked as important factors contributing to the development of resistance mechanisms. It is clear that the use of wax free from pesticides can reduce the undesirable selective effect that these resistance mutations can exert on varroa populations (Calatayud-Vernich et al.^2018^; Vlogiannitis et al. 2021a, b).

It is considered essential to conduct intensive research to reveal the mechanisms by which the accumulation of acaricides in beeswax, honey, and bee bread affects the development of resistance in varroa populations, especially regarding the subject of this study, pyrethroid-based acaricides. Based on the results obtained, the relationship between mutations and pyrethroid resistance supports numerous studies. However, it is important to note that the data were obtained from a limited number of mites collected from specific geographic regions. The rational use of pesticides in the fight against *V. destructor* is the shared responsibility of the entire beekeeping industry, including public institutions, universities, industry, and civil society organizations. However, coordinated action is required to prolong the effectiveness of treatments and delay the development of resistance mutations that threaten the sustainability of beekeeping.

## Data Availability

No datasets were generated or analysed during the current study.
